# 

**Published:** 1883-04

**Authors:** 


### BOOKS RECEIVED.

**Transactions of the American Medical Association.** Volume 33, 1882.

**Medical and Surgical History of the War.** Part iii, vol. 2.

